# Design and Field Test of a WSN Platform Prototype for Long-Term Environmental Monitoring

**DOI:** 10.3390/s150409481

**Published:** 2015-04-22

**Authors:** Mihai T. Lazarescu

**Affiliations:** Politecnico di Torino, Dipartimento di Elettronica e Telecomunicazioni, Corso Duca degli Abruzzi 24, I-10129 Torino (TO), Italy; E-Mail: mihai.lazarescu@polito.it; Tel.: +39-011-090-4111

**Keywords:** WSN, IoT, pervasive environmental monitoring, long-term operation, low maintenance, low-cost WSN nodes, low-cost WSN deployment, protocol design, low power

## Abstract

Long-term wildfire monitoring using distributed *in situ* temperature sensors is an accurate, yet demanding environmental monitoring application, which requires long-life, low-maintenance, low-cost sensors and a simple, fast, error-proof deployment procedure. We present in this paper the most important design considerations and optimizations of all elements of a low-cost WSN platform prototype for long-term, low-maintenance pervasive wildfire monitoring, its preparation for a nearly three-month field test, the analysis of the causes of failure during the test and the lessons learned for platform improvement. The main components of the total cost of the platform (nodes, deployment and maintenance) are carefully analyzed and optimized for this application. The gateways are designed to operate with resources that are generally used for sensor nodes, while the requirements and cost of the sensor nodes are significantly lower. We define and test in simulation and in the field experiment a simple, but effective communication protocol for this application. It helps to lower the cost of the nodes and field deployment procedure, while extending the theoretical lifetime of the sensor nodes to over 16 years on a single 1 Ah lithium battery.

## 1. Introduction

Since the Internet of Things (IoT) paradigm was first coined more than a decade ago [1], wireless sensor networks (WSNs) were included among its enabling technologies as flexible, low-cost systems suitable to build dense and cost-effective monitoring infrastructures [2]. WSNs have relatively low material and deployment costs, since they do not need wiring, can self-configure and can operate for extended periods of time with very low, if any, scheduled maintenance. Sensor networks are also considered among the best techniques for environmental data collection for a variety of open-space applications, either close to human daily activities or remote, in the wild.

WSNs are typically made of multiple nodes, which are small and portable embedded computing systems interfaced with specialized transducers and radios for short-range communication. They are capable of autonomously monitoring, processing and transmitting various parameters at diverse locations for extended periods of time, using very limited energy and often without any maintenance in their lifetime.

More than a decade after the WSN vision, the application domain experts expect the technology to solve application demands inexpensively and with low effort. However, the multidisciplinary challenges raised by WSN optimization to multiple application needs have been constantly acknowledged over the past decade, especially for outdoor applications [3–5]. Most networks remain relatively small in size and/or are deployed for short periods of time, and node programming often requires experienced software engineers [6]. High cost and perceived low reliability are still important barriers [7], out of which maintenance cost may significantly increase in the case of remote deployments or in locations that are difficult to access.

As for most maturing technologies, WSNs reached the stage where standards consolidate most existing achievements. IEEE 802.15.4 and 6LoWPAN set the groundwork for applications with IPv6 support. Software-defined radio efforts build on these, aiming at more robust and versatile communication architectures through antenna-, frequency-, modulation- and data-rate diversity, on which services for diverse application spaces can be built.

At the same time, environmental monitoring has constantly grown in importance. Routinely collected data about the state and the evolution of the environment can help to detect and alert of hazardous events and to assess and optimize the long-term sustainability of, e.g., population growth, urban sprawl, invasive species, climate change and habitat destruction.

Nevertheless, the outdoor monitoring application domain is often challenging [8]. The quality of radio communication between nodes can be unpredictable, especially during atmospheric events (e.g., heavy rain, snow, frost, fog), due to plant and foliage growth or their movement due to wind, or due to the accumulation of dirt or insect nests on nodes. Repeated mechanical stress induced by large daily temperature variations may prematurely break the nodes, increasing the operating cost, since maintenance in open nature is usually difficult. Communication protocol scalability and the cost of nodes and field deployment may be stretched for applications that require thousands or tens of thousands of nodes for pervasive monitoring.

WSN platforms typically rely on existing hardware and software components to support a broad range of applications, often using some cross-layer optimizations. However, applications with stringent requirements, such as low cost, a large number of nodes, long service time, fast field deployment and reduced maintenance, can motivate custom design and optimization of the entire WSN platform.

In this paper, we focus on the needs of a demanding application, pervasive wildfire monitoring using *in situ* distributed temperature sensors. Early detection of forest wildfires is very important for fire fighting success and to effectively limit damage and cost. We comparatively analyze existing detection and alert solutions and define the design objectives of a WSN platform that can overcome most of their weaknesses. Then, we present the main design phases, the effort spent, the major pitfalls building the first prototype and the lessons learned from its field test. We also show how the resulting WSN platform can support a large class of environmental monitoring applications, increasing its value and reducing its return on investment.

Platform specifications were defined in collaboration with an industrial beneficiary and respond to pragmatic and stringent requirements for pervasive wildfire detection and alerting. For this purpose, the company consulted several actors in areas subject to wildfires, such as firefighters, state forestry corps, civil protection and forest rangers.

The main contributions of the paper can be summarized as follows:

(1) A methodical holistic analysis of the effects of the application requirements and of the available design options for the platform hardware, software, communication protocols and field deployment procedures on the overall platform performance, the cost and the reliability of its nodes, of the platform commissioning and exploitation. This method can be applied for the analysis of other WSN platforms in the context of different application requirements.
(2) Design criteria, optimization and field validation of cost-effective sensor nodes and gateways that use much lower resources and energy than the typical nodes in the literature based on off-the-shelf components.
(3) Analysis, definition and field test of a cost-effective and reliable field deployment procedure for the network nodes that minimizes human-introduced errors.
(4) Design, optimization and field test of a simple, yet effective field communication protocol that matches application requirements well, significantly contributes to the reduction of node energy consumption and cost and supports an effective field deployment procedure for the nodes. It can also scale up to support networks with a very large number of nodes.
(5) Analysis and selection of the hardware and software features to implement on node prototypes designed for laboratory testing in order to adapt them, in a short time and with reduced effort, for a relatively long, unattended field experiment with two equally important purposes: to check and demonstrate the autonomous operation of the platform and to acquire sufficient visibility of node and system behavior that can be used to improve the design of the platform.

The rest of the paper is organized as follows. Section 2 reviews related published works and defines the specifications of the system through comparative analysis of the strengths and weaknesses of existing methods. Section 3 outlines the design criteria, which drive the platform design decisions. Section 4 explores the most important design decisions made for the implementation of the WSN platform components. Section 5 presents how the prototype platform was prepared for a field test, on short notice. Section 6 presents the unfolding of the field test, the major issues during its various phases and the most important lessons learned to improve the WSN platform. Section 7 presents a summary of the future work and concludes the paper.

## 2. System Definition

To better define the specifications of the proposed wildfire monitoring method, we will briefly review several of the most representative existing techniques with a focus on cost, detection reliability and effectiveness for early alerting.

### 2.1. Wildfire Detection Methods

Wildfires are quasi-natural hazards likely to occur under specific climatic, weather and vegetation conditions [9]. A small fraction ignite from natural causes that include lightning, volcanic eruption, sparks from rockfalls and spontaneous combustion, but most ignite from human activities, such as arson, discarded cigarettes, sparks from equipment, power line arcs and controlled field burning [10–12].

While most common wildfire causes may vary throughout the world [13,14], early detection is always very important, because fires may spread fast and escape firefighters' control, potentially causing significant economic damage, threatening human lives, homes and resources and devastating wildlife, habitats and ecosystems [15].

Wildfire frequency and devastation make the continuous monitoring of the areas at risk one of the most well-known environmental monitoring activities. Although many detection techniques were proposed, cost-effective and reliable early wildfire alerting is still difficult to achieve.

#### 2.1.1. Ground-Based Remote Detection Methods

Traditionally, wildfire monitoring is based on continuous human surveillance from selected vantage points in the field attempting to recognize smoke patterns during the day or the light from fire flames during the night using visible light [16,17]. The authors argue in [18] that observers' reports are often insufficient to define an effective firefighting strategy and that the field video surveillance monitored in the operational center can provide richer, actionable information and increase the surveyed area by the operator. However, since remote field monitoring increasingly subjects the operators to fatigue, boredom and loss of concentration, several semiautomated wildfire recognition systems are proposed [19–34]. These systems reduce the effort of the analysts, which basically need to validate the positives triggered by the automated detection methods [22].

A class of detection methods look for combustion-generated heat using infrared (IR) imaging. The equipment is rather expensive, has reduced spatial resolution and is prone to false positives [21,23]. Accuracy can be improved by automated cross-matching of IR images with visible or ultraviolet (UV) ones or with meteorological and geographical data using various processing and decision making techniques, but at a higher equipment and maintenance cost [24].

Optical spectrometry can detect combustion-specific products in smoke [25,26] by analyzing its sunlight absorption patterns. It has good reliability, but is limited to scans above the horizon (thus, for smoke that clears the tree line), and at night, it switches to the less effective flame detection [27]. Strong winds may lower or disperse the smoke, delaying detection. However, wind significantly increases the speed of fire spreading; thus, early alerting of firefighters becomes crucial for blaze containment.

Light detection and ranging systems (LiDAR) analyze the laser light backscattered by smoke [28,29]. High-power laser beams may be expensive to produce, although, recently, researchers proposed lower cost techniques [30], and they can also be a public health hazard. Its effectiveness may be limited by obstacles in the line-of-sight, fog or low altitude clouds, which are a likely occurrence in mountain climate.

Radio-acoustic sounding detection (RASS) can detect crown and surface wildfires from thermal maps built by inferring air temperature from shifts in the propagation time of acoustic waves [31]. The system can account for atmospheric events, like wind and some static echoes from vegetation or landscape, but it requires a clear line-of-sight right above the tree tops for proper operation. Moreover, its deployment and calibration can be complex.

Remote detection systems tend to generate false positives (especially those based on visible light image analysis) and need the support of human analysts. Those that are more accurate are also more expensive due to higher equipment and maintenance cost [35].

#### 2.1.2. Overhead Remote Detection Methods

Satellite-based wildfire detection can reach low false alarm rates in near real time. However, geostationary satellites may have limited spatial resolution, while polar orbiting satellites have limited temporal resolution, which can be significant for rapidly-evolving wildfires [36,37]. The accuracy of the automated methods based on satellite monitoring can be further reduced by image acquisition problems and image navigation errors, which can compromise the monitoring of individual fire events that would otherwise be correctly identified by analysts [38].

Unmanned aerial vehicles (UAV) can efficiently monitor for wildfires over large areas [39,40]. Equipped with various fire detection techniques, they can survey the area continuously and cost effectively from a diversity of vantage points, much closer than the satellites. UAVs operation can be affected by local weather conditions (fog, strong wind, low altitude clouds), wind being a well-known risk factor during their takeoff and landing [41].

These methods also share most operating limitations of the ground-based remote detection techniques mentioned in Section 2.1.1, like unfavorable weather, obstructions of the direct line-of-sight, ambient light quality and incidence angle.

#### 2.1.3. Pervasive *In Situ* Detection Methods

*In situ* pervasive wildfire detection has several advantages over remote detection [42]. First, the sensors are closer to the events (e.g., combustion); thus, their sensing is generally less susceptible to interference from external factors (e.g., wind, fog, obstructions). Second, *in situ* detection is implicitly redundant to some extent, since the higher sensor density makes it likely that more than one of them detects an event within a short time window. Nevertheless, natural occurring, non-fire-related phenomena (such as fog, dust or insect nests) may still trigger false alerts, even for pervasive methods, or the effectiveness of some detection methods may be limited at night or in low visibility conditions.

Moreover, it is important to note that the cost per unit of surface of *in situ* monitoring is usually higher than that of remote sensing, both for the initial deployment and for the maintenance [43]. Thus, reducing these costs can significantly extend the application range that can be economically addressed using pervasive methods. Their finer spatial and temporal resolution and the generally higher detection accuracy can complement the remote detection methods and can be justified for areas with higher risk of fire or where humans or goods are more exposed, such as along roads or trails that cross forests or at the interface between human habitat or resources and forests.

WSNs are among the best-suited systems for pervasive *in situ* long-term environmental monitoring, even in wild and harsh conditions [44–50], although other techniques have been reported, as well, e.g., based on optical fiber [31]. WSN nodes typically include the transducers and processing power to autonomously detect specific fire effects within their range, such as light, smoke, heat and noise [51–55]. The nodes are deployed within the area to monitor using application-specific criteria [56] and periodically check for fire signatures using their on-board sensing and processing capabilities. The control center is notified of positive matches by using radio messages sent either directly to gateways or forwarded through a mesh network made by their neighboring nodes.

Most fire detection techniques used by pervasive WSNs are shared with the remote methods (see Sections 2.1.1 and 2.1.2) with various degrees of detection confidence, power profile and cost [49,57]. Low-power techniques are preferred, since the limited sensor node energy is an important selection factor, energy being a direct and indirect contributor to WSN solution cost (we will analyze this in detail in Section 3.1.1).

Techniques based on low-power image processing [58] or radio-acoustic detection of the sound of burning fire [31] performed on board the sensor nodes are reported, but they generally require higher processing energy. Detection of infrared [59] or ultraviolet radiation [60] that are correlated with fires have a lower energy consumption profile and can be used alone or in conjunction with smoke detection for increased accuracy. Smoke particle detection can be combined with temperature sensing [53] (either as absolute value or variation speed). The detection of temperature patterns can be used alone (and this is arguably one of the most cost- and energy-effective techniques) [61], or correlated with humidity readings [44], or with humidity, wind and rainfall data that are periodically retrieved from the application server [48]. In general, techniques based on data fusion from multiple transducer types can increase detection reliability [35,62,63]. Reliability can be increased also using adaptive methods on sensor nodes [64,65] or on sensor and gateway nodes, the latter benefiting from data aggregation from multiple sensor nodes [66].

Other methods are based on the detection of abnormal levels of combustion gases, standalone or combined with temperature detection [67]. However, gas transducers may require higher power and periodic recalibration. WSN detection can also be combined with UAV monitoring [50] to increase the detection accuracy and to collect the necessary data to define and coordinate the actions of the firefighters. While most WSN deployments are designed for long-term monitoring, they can be deployed in the affected areas for the short term to continuously collect data to help support firefighters' operations in the field [68].

Meeting the stringent requirements for effective long-term outdoor environmental monitoring is generally challenging for WSN technologies [69–71]. Nodes need to reliably operate for very long times without or with minimal maintenance in variable and sometimes extreme climatic conditions. Outdoor applications demanding a high number of nodes put additional emphasis on node cost and reliability. Node weight, size and ruggedness become also important for deployments in fields that are difficult to reach, e.g., when nodes are transported in backpacks [8].

### 2.2. System Specifications

The analysis in Section 2.1 shows that *in situ* pervasive sensing is among the fastest and most reliable monitoring methods. A low false alert rate due to higher detection reliability can reduce the need for human analysis and the effort of the field personnel, thus reducing the exploitation cost. Earlier fire detection can help the firefighters to contain the risks and damages, increasing safety and reducing costs.

WSN technology looks well suited for distributed sensing and reporting, but to be economically viable, it needs to scale well for large deployments and to have reduced initial and operation cost: low sensor node cost, reliable operation for extended periods and to require very few or no maintenance at all.

Transducers on board the WSN nodes typically use energy sparingly to preserve their reserves and life time. Among the effects of the fire, heat is always present, and high temperatures are among its most devastating effects. The temperature of the surrounding air can be energy- and cost-effectively measured (e.g., using inexpensive negative temperature coefficient (NTC)transducers), and the WSN nodes can periodically sample it looking for abnormal temperature patterns that are highly correlated with convection heating from fires. For example, the nodes can alert when the absolute temperature value or its rate of increase rises above specific thresholds [53]. The thresholds should be set high enough to prevent false alerts due to naturally occurring temperature patterns and as low as possible to improve detector sensitivity and timely alerting.

As discussed in Section 2.1.3, fire detection accuracy can increase by combining measurements from different types of transducers, each sensitive to different effects of the fire. For instance, abnormal air temperatures can be correlated with the levels of infrared radiation on wavelengths closely related to the presence of fire, for the same monitored area.

Transducers of different types can be mounted on the same sensor node or on different, specialized nodes. A node with multiple transducers can correlate the measurements on board, reducing network traffic. However, multiple transducers also increase the node cost, energy consumption and resource requirements. They may also add deployment constraints, increasing its complexity and cost.

**Figure 1:**
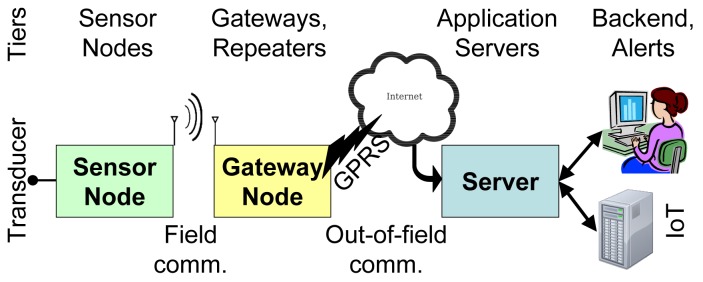
Typical tiered structure of WSN platforms.

Specializing the nodes by transducer type allows one to differentiate their deployment requirements to optimize their operation. For instance, nodes that sense infrared radiation can be attached to the underside of the lower branches of the trees, facing down, at heights of a few meters above the ground, while the temperature sensors can be attached on tree trunks, at 0.5–1 m from the ground, to have good thermal contact with the hot air rising from nearby fires. The deployment density may also differ by node type. Field data from nodes can be correlated upstream, e.g., on the gateway or server for a typical tiered structure, like the one shown in Figure 1.

Application-specific trade-offs are usually made between the required detection accuracy and the implementation cost. The industrial partner initially considered fire detection solutions based on low-cost sensors, which consume no energy in standby mode and that are activated by fire (e.g., heat-activated power supplies [72] or conventional small batteries that supply the node through a bimetallic switch only when heated above a threshold [73]). However, the quality of service (QoS) cannot be monitored for these solutions, thus they cannot be considered reliable in the general case.

Consequently, the specifications evolved towards more typical WSN platforms that use active sensor nodes and a tiered structure as shown in Figure 1. Sensor nodes are fit with transducers to sense the environment properties of interest and can communicate with the gateway (or sink) nodes using short-range in-field radio communications, either directly or through multihop message forwarding. The gateways may further process and aggregate the data and can communicate with the application server over long-range data channels. The server has many functions, such as reliably storing the field data, providing suitable user interfaces for observation and analysis, data post-processing, configurable alerts and interfacing with other IoT applications.

From the comparative analysis in Section 2.1, the requirements of a WSN platform well suited for reliable and cost-effective long-term pervasive environmental monitoring can be defined as follows:

low-cost, small-sized nodes (sensors and gateways) that can process the transducer data on board, perform self-tests and recover from errors (preserving field data if possible);
receive software updates over the air with minimum service disruption and have one or more long-range communication channels (at least the gateway nodes);
the field communication protocol should efficiently support:
  – network sizes from a few sparse nodes up to a very large number of nodes;
  – low data traffic per node, in small packets;
  – resource-constrained reliable operation, with small memory and energy overheads;
  – reliable operation in case of node failures, especially if several nodes are destroyed by fire;
transmit events instead of raw data to reduce communication energy by processing the data close to the source (on sensor nodes and gateways);
fast and reliable field deployment procedure for network nodes;
adequate gateway hardware and software resources and interfaces for application-specific extensions (e.g., local transducers, data storage, communication channels, other peripherals);
node ruggedization to withstand long-term exposure to environmental conditions;
long maintenance-free service (the beneficiary requirements were 5–10 years for the sensor nodes);
high availability of service for nodes and servers;
multimodal access to server data for human interfaces and automated processing;
programmable multichannel alerts on field events;
automatic detection and reporting of platform faults;
extensible server architecture for integration in IoT applications.

These specifications would also allow the reuse of the platform for several event-based long-term applications besides wildfire monitoring, such as:

water level of lakes, streams, sewage. Sensor nodes can detect and alert about extreme levels (high or low) and periodically report level statistics;
gas concentration in air for laboratories, deposits, ship holds, cities. Sensor nodes can detect potentially harmful concentrations of one or more gases;
soil properties for precision agriculture;
static or dynamic parameters for structures, e.g., the inclination of dams;
position changes, e.g., land slides;
lighting level, e.g., for intrusion detection in dark spaces, like cable channels;
infrared radiation from combustion (fire) or human or animal presence, for security or habitat monitoring. On-node analysis using thresholds and speed of variation can screen alerts from natural-occurring patterns.

Platform reuse can improve the economic sustainability of its development and maintenance. This is important, since cost can be a major factor that prevents commercial adoption of *in situ* pervasive monitoring solutions.

The overall cost of the platform has several components that we consider in detail during platform design in Section 3:

Node cost, which is roughly proportional to the monitored area and can significantly weigh on the total cost. Generally, a WSN has fewer gateways than sensor nodes; thus, platform design needs to carefully optimize at least the cost of the latter.
Exploitation cost basically made up of the cost of the analysts to validate the alerts and the cost of node and network maintenance. The rate of false alerts can be kept low through several techniques: performing the sensing close to the event source, using selective sensing methods less susceptible to interference from unrelated events and using data fusion from multiple sensor types [74]. Maintenance may be needed to periodically clean the nodes to restore their nominal sensing or energy harvesting levels or to replace exhausted batteries. This can be reduced by using maintenance-free transducers and low-energy long-life sensor nodes. Nodes can fail for various reasons, including mechanical stress due to daily temperature variations or extreme climatic conditions. Adequate node hardware design and ruggedization should minimize the effects of long-term environmental exposure.
Deployment cost, which can significantly increase when the monitored area is difficult to access or if deployment errors are discovered later, during network exploitation, requiring additional field expeditions to correct them. Deployment cost depends also on other factors, such as the complexity and time needed for the deployment of the network nodes, the level of specific knowledge expected from the deployment personnel and node size and weight (if they are manually transported in field).

Most platform costs can be reduced through optimizations of the design of its components with a holistic view of the full system, as will be shown in Section 3.

## 3. Platform Design Criteria

We will present in the following the most important node design considerations and decisions based on the specifications in Section 2.2.

### 3.1. Sensor Node Design Criteria

WSN platforms for pervasive *in situ* monitoring may need to deploy many sensor nodes, which can affect several cost components of the platform. In the following, we will analyze the design options for the major contributors to sensor node cost, as well as their contributions to the various components of the platform cost that were presented in Section 2.2.

#### 3.1.1. Sensor Node Energy Supply

Energy supply can be an important cost of the sensor nodes. Its role is to provide energy for node operation between maintenance (e.g., for battery replacement or node cleaning), with the continuity necessary for the expected node quality of service (QoS).

An important decision is whether to use a regenerative supply. Energy harvesting can be effective when ambient energy is available, such as vibration, fluid flow, temperature differences and light [75]. Although their cost and QoS are difficult to estimate in the general case, combined energy harvesting can increase the availability [76]. These supplies may add to node or platform cost, for instance by increasing the physical dimensions of the node, by additional casing requirements (e.g., a waterproof transparent window for light harvesting), by additional restrictions for field placement for effective ambient energy harvesting or by increasing the complexity of the deployment procedure.

Primary batteries, on the other hand, store all of the energy needed for the entire node lifespan or the period between scheduled maintenance. The minimum battery capacity can be obtained by multiplying the node average energy consumption and the expected lifespan. Battery cost is roughly proportional to its capacity, but the scheduled maintenance for replacement of depleted batteries can be a major contributor to the overall solution cost.

#### 3.1.2. Sensor Node Communication Protocol and Radio Device

Radio communication is widely recognized as one of the major energy drains on sensor nodes. Thus, it influences the selection of the energy supply (with the criteria presented in Section 3.1.1) and some other node costs that we will analyze in the following.

The largest part of the communication energy is typically spent by the receiver while listening for incoming data packets. For this reason, most communication protocols operate the receiver with very low duty cycles (typically 1%–3% for random MACs [77,78] and much less for synchronous MACs [79]). Even more, the receiver can be eliminated altogether for random access unidirectional communication in star topologies.

Multihop message forwarding typically uses less energy to cover the same distance and may be less affected by node faults or changing propagation conditions. However, mesh networks require more microcontroller resources and involve regular packet exchanges to maintain network connectivity. These may increase both node energy consumption and cost. Moreover, message routing in large mesh networks may be affected by dynamic effects that are hard to detect, such as bottlenecks or instabilities [8,80,81], which may increase deployment complexity and cost. Synchronous protocols also need an accurate timer constantly running on the nodes, which further increases their cost and energy consumption.

Transmit-only nodes in star topologies can use very small protocol stacks and have much more relaxed requirements for on-node timing accuracy. Thus, they need less microcontroller resources and energy, but they cannot create mesh topologies to improve robustness and reduce transmit power. Furthermore, these nodes cannot actively avoid message conflicts or receive confirmations, queries, configuration changes or in-field updates for node software.

#### 3.1.3. Sensor Node Functions

Besides the main function of environment sensing and processing, sensor nodes can implement functions that improve the overall system reliability and cost.

Periodic node self-test and error recovery functions increase the reliability and QoS of the monitoring platform and reduce maintenance requirements and cost. Nodes that aggregate peer messages before forwarding should implement suitable redundancy policies to prevent data loss if they reset themselves in order to recover from errors or if they fail.

Normally, the sensor nodes do not have user interface elements, such as buttons, LEDs and displays, which can increase the bill for materials, the ruggedization cost and the energy consumption. Thus, for interactive operations, like checking the quality of an installation position in the application field or retrieving their ID, the nodes should provide the operator simple, yet effective means to selectively address and communicate with them while in close physical vicinity. Moreover, specific node self-testing and reporting functions should be used at the end of the production line to lower production cost.

However, additional functions increase the complexity of the node program, thus the probability of defects [82], which can adversely impact the overall system reliability and increase the maintenance cost. Programming the nodes using high-level, clean, application-optimized code and component reuse may require less resources and energy, thus reducing the cost and improving the reliability of the node.

#### 3.1.4. Sensor Node Hardware Components

In the following, we examine some criteria that guide the sensor node hardware selection.

Smaller bills for materials generally increase the production and operation reliability and reduce costs.

Transducer selection closely depends on the detection method. For instance, fire can be sensed through smoke or heat, and the latter can be sensed by temperature (e.g., using an NTC) or by radiation (e.g., using passive infrared transducers). Low-cost and power-efficient transducers, which can operate on very low duty cycles and with less energy, can significantly reduce node and maintenance cost.

Radio devices with a baseband modem may reduce the software complexity and energy consumption.

The microcontroller resources should fit the application needs closely, since oversizing it may increase the cost and energy consumption on most power levels.

### 3.2. Gateway Design Criteria

In typical WSN deployments, there are significantly fewer gateways than sensor nodes. Hence, the node cost of the gateways contributes less than the cost of the sensor nodes to the overall cost of the WSN platform. However, the features of the gateways can indirectly influence the cost of the platform in several ways, as we will show in the following.

#### 3.2.1. Gateway Communication

The gateways typically have two types of communication capabilities: long range and short range. The long-range communications are used to connect with the application server either directly or through the Internet. The short-range communications are mainly used to connect with the field nodes, either sensor nodes or peer gateways.

Long-range communication can use several technologies. A cellular data network is commonly used in outdoor fields that are covered by mobile data services [83]. Alternatively, dedicated communication links on either a private or public radio spectrum can be used, e.g., the 868-MHz short range devices (SRD) band allows ranges up to 40 km [84], and long-range Wi-Fi links can exceed 100 km [85]. Satellite links may also be used [86], especially for remote, isolated fields.

The constraints for the short-range in-field communications are less stringent for the gateways than for the sensor nodes (which were discussed in Section 3.1.2). The gateways have more energy reserves and computing resources, thus they can support most communication protocols: from simple low power listening (LPL) for random access MACs in star topologies and up to complex routing and synchronization schemes.

Thus, the gateways can help reduce the overall cost of the WSN application by supporting the short-range communication protocol that optimizes the cost of the sensor nodes, which is a significant component of the overall cost of the solution.

#### 3.2.2. Gateway Energy Supply

Long-distance communication of the gateways typically requires much more energy than the short-range in-field one of the sensor nodes. Gateways also have more hardware resources, such as larger program and data storage capacity, more powerful microcontrollers or microprocessors and accurate, always-running timers. All of these combined require significantly more energy than the sensor nodes.

Thus, the gateways generally have higher energy reserves and may also have ambient energy harvesting devices to increase their lifespan between scheduled maintenance (which may be needed for, e.g., the replacement of the (rechargeable) battery or for the cleaning of the energy harvesting devices). Even if the gateways may require scheduled maintenance more often than the sensor nodes, its cost may be acceptable: a WSN deployment for pervasive environmental monitoring typically has fewer gateways than sensor nodes, and the gateways are generally deployed in locations that are more accessible than the sensor nodes (see Section 2.2), which further reduces the cost of gateway maintenance.

Given that gateway energy reserves are far larger than those of the sensor nodes and that gateway maintenance is less expensive than the maintenance of the sensor nodes, it can be cost-effective at the network-level to bias the energy consumption towards the gateways if this helps with reducing the cost of the sensor nodes and their scheduled maintenance requirements.

#### 3.2.3. Gateway Functions

Besides the main function to collect and forward to the application server the field data from the sensor nodes (and from the on-board sensors, if any), the gateways can implement other functions that improve the overall system reliability and cost.

The gateways should prevent the loss of the aggregated field data in case of non-fatal hardware faults or of self-resets due to software defects. Moreover, in case of repeated or fatal software errors, the gateways should fall back to a safe operation mode, which allows remote debugging or recovery to reduce service interruptions and the cost of in-field maintenance.

During field deployment, the gateways should assess the suitability of the installation position in terms of network reachability and link quality with the other nodes and the server, as well as the effectiveness of the energy harvesting (if available).

### 3.3. Server Design Criteria

The main purpose of the application server is to receive field data and store them reliably for later access upon request. It bridges the low-power communication segments (that operate under strict latency-energy trade-offs) with the fast and ubiquitous data access needed by human interfaces or IoT applications. As shown in Figure 1, the server provides interfaces for field nodes, operators and supervisors of the field, alert channels and for external systems.

For a high QoS, the server should implement high-availability techniques, e.g., a secondary server should be kept in sync and automatically take over the service if the main server fails.

The protocol between the nodes and the server should be simple to reduce the resource requirements for the nodes and, as much as possible, stateless, because the long-range connections at the fringe of mobile data coverage can be often unstable and the overhead for resuming an interrupted transfer should be minimized. For the same reason, the protocol should provide acknowledgments for data reception, and these should be sent only when the data are successfully replicated on the secondary server to avoid field data loss due to single points of failure.

Gateways that need to enter the fail-safe operation mode may use a communication protocol with the server that is optimized for the operations executed in this mode (e.g., software update). The server should implement this protocol and the associated operations, as well as promptly notify the operators when a gateway connects using this channel, to take corrective actions.

## 4. Platform Design

Platform design aims at a cost-effective implementation that closely follows the specifications in Section 2 and the design criteria in Section 3.

Considering the multi-objective optimization needed to satisfy sensor node specifications and design criteria, we decided for a custom design and development of communication protocols (both for in-field communications and with the server) and node hardware and software.

In the following, we describe the design of the prototype platform, the field test preparation and unfolding, the lessons learned from the test and how we can use them for improvements.

### 4.1. Communication Protocol Design

The communication protocol can affect the performance of a WSN platform in several important ways. First, the protocol is mostly responsible for propagating the data across the network according to application requirements. Consequently, it controls the most part of the radio activity of the nodes, which is generally recognized as a major energy consumer. Thus, the protocol can significantly influence the selection of energy supply type and size, which relate closely to its cost and to the scheduled node maintenance. Moreover, the characteristics of the communication protocol may also affect the complexity and quality of the field deployment of the network, thus its duration, cost and the need for subsequent corrective network maintenance [6].

Most WSN deployments tend to use simple communication protocols that are enough to satisfy the requirements of the applications [87]. However, very few appear to consider during network design the effects of the protocol on the complexity, quality, maintenance requirements and cost of network deployment [43]. Deployment and deployment-related maintenance can add up to significant costs for pervasive environmental monitoring applications.

The analysis in Section 3.1.2 presents some of the most significant trade-offs to consider when selecting the protocol for a specific application. For instance, using a radio receiver can increase the cost of the sensor node and of the application by adding to the cost of components, of the microcontroller (may require more resources), increases node energy consumption, thus adding to the cost of energy supply and/or the scheduled maintenance to change node batteries. However, a receiver also allows a better management of the communication channel, can increase the reliability of message delivery, can reduce the transmission power per node through multihop message forwarding and can support bidirectional network communication that allows one to remotely query or reconfigure the nodes in the field.

To define the specifications of the communication protocol, we started from the analysis of application requirements. The communication needs of sensor nodes by state can be summarized as follows:

normal state: the node periodically communicates its health status and, if possible, some background environmental measurements;
fault state: the node communicates the nature of the fault (if the fault does not affect the radio); alert state: the node detected a fire and reports the event in the shortest time, with the highest priority.

If the nodes periodically communicate their health status and some background environmental measurements, remote node interrogation becomes less important or even impractical for a large number of nodes, which are typical for pervasive monitoring methods (from hundreds to tens of thousands of nodes per application). Thus, for such applications, a bidirectional communication may be less important than the optimization of node cost.

Multihop message propagation through the network using a mesh topology can reduce the transmit power of the nodes, which may lead to lower energy consumption at the node level. However, two other factors should be considered. First, operating a radio receiver, even with very low duty cycles, can still significantly raise the energy consumption of the node. Second, lower radio frequencies, which propagate better in forest environments [88,89], have also a considerably longer communication range for the same transmit power, thus making the use of a star topology more practical. Moreover, a network with a star topology can be functional, even with sensor nodes with no receive capabilities, which can lead to multiple cost reductions, as we have shown.

Thus, on the one hand, we decided to use the 433-MHz UHF industrial, scientific and medical (ISM)band for its better propagation in forests. On the other hand, we analyzed if a communication protocol based on unscheduled channel access can satisfy the requirements of the application.

Communication protocols with unscheduled channel access are well known in the literature [77]. In the following, we will analyze if we can completely remove the receiver from the sensor nodes to achieve multiple cost reductions.

Without receive capabilities, the sensor nodes have no means to know if a message reached its destination or was lost. Thus, sufficient communication redundancy should be built into the protocol to keep message loss to acceptable levels for the application. It should be noted that the application is more tolerant to the potential loss of low-priority periodic health status messages than to high-priority and delay-sensitive fire alerts.

Higher transmission redundancy may increase the delivery probability of the messages, but it also increases the energy consumption of the node and the probability of collisions. Thus, protocol design should consider the effects of redundancy on message loss and on the delay of the detection of faulty nodes that are no longer able to transmit their status, for various network sizes.

For this purpose, we have set up a network simulation model in SystemC [90] as follows. The sensor nodes were modeled as transmitters that periodically send a health status (heartbeat) message. The transmission period is affected by a static error that is initialized randomly in the range +10–+30%, corresponding to the error range of the watchdog timer of the microcontroller that was used to control the timing on the sensor node.

The gateway node was modeled as a receiver implementing LPLto listen for incoming messages from the sensor nodes. The wakeup period for RF sampling was set slightly shorter than the length of a message, 0.3 s (including the preamble). Normally, the radio receivers can correctly decode the strongest of two overlapping messages if the amplitude difference exceeds the receiver signal-to-noise ratio, thus losing only one of the two. The same stands for messages that overlap only partially over the preamble. However, for the purpose of the simulation, we opted to pessimistically consider that two overlapping messages are both lost, regardless of the amplitude ratio or the extent of the collision.

**Figure 2:**
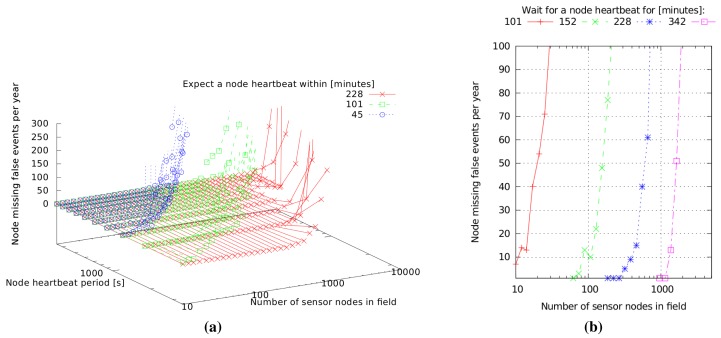
Simulation results of the field communication protocol for design space exploration. The rate of the missing node erroneous reportsincreases with the number of nodes and with the heartbeat period and lowers with the increase of the heartbeat listening window of the gateway (**a**); missing nodes can be detected in less than 6 h with a negligible rate of erroneous reports, for a 1 h heartbeat period (**b**).

Figure 2a shows the simulation results for a year of operation for fields with one gateway that receives heartbeats from 10–5000 nodes. The node heartbeat period varies from six minutes to one hour, and we count the number of times that a sensor node is declared missing over one year of operation for different time windows in which the gateway expects to receive at least one heartbeat from a node (to avoid cluttering Figure 2a, we have plotted only the results for three windows, of 45, 101 and 228 min).

We see that for a given heartbeat period and gateway window, the erroneous node missing events increase with the number of nodes. This is expected, since more nodes randomly sending heartbeat messages increase the probability of collisions, thus of message loss.

We also note an increase of erroneous node missing events with the increase of the sensor node heartbeat period for a given number of sensor nodes in the field and a given gateway window. This result can be counterintuitive, since longer heartbeat periods mean less concurrent messages contending for the communication channel on average, thus lower collision probability. However, considering that the gateway window for receiving a heartbeat message from a sensor is constant, a longer heartbeat period means also that fewer heartbeats are sent within the window, which effectively decreases the protocol redundancy that is needed to overcome message losses typical for unscheduled MACs.

We used these simulation results to choose a compromise between the frequency of sensor node heartbeats (which contribute to node energy consumption), the delay after which the gateway detects missing nodes and the frequency of false node missing reports. As we will show in Section 4.2, one heartbeat per hour increases node energy consumption by about one third, which we consider acceptable for this application. Furthermore, in Figure 2b, we see that for a network traffic made of one heartbeat per hour per sensor node and a gateway window of slightly less than six hours, we achieve very low false positives for fields up to about 1000 nodes. This is realistically large, since we expect at most a few hundred nodes per gateway in practical deployments. Furthermore, a six-hour delay in reporting missing nodes is considered suitable for scheduling network maintenance operations.

Thus, the main characteristics of the communication protocol can be summarized as:

under normal conditions, the sensor nodes send one heartbeat per hour;
fire alert messages are sent with much higher frequency (every few seconds) to ensure their timely propagation, even at the cost of losing concurrent heartbeat messages. The communication channel capacity can support the alert traffic well, since, assuming no collisions, 1000 nodes sending one 0.3 s heartbeat message per hour each occupy at most 8.3% of the channel capacity;
the gateways can detect a missing node in less than six hours.

This simple protocol satisfies the requirements of the application well. It does not need a receiver on the sensor nodes, which primarily lowers node cost and energy consumption. Moreover, without a receiver, the operation of the sensor nodes is defined only by their own programming. This means that the energy consumption for each node in normal conditions is predictable, and we calculate it in Section 4.2. In the alert state, the node will spend much more energy in order to propagate in a timely manner the alert messages to the gateways, which can significantly reduce its energy reserves. However, this is not an issue, since the heat of a fire will very likely destroy the node shortly after detection.

### 4.2. Sensor Node Design

**Figure 3:**
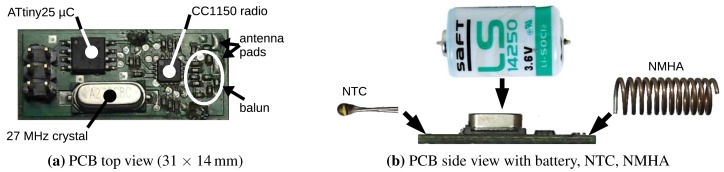
Sensor node prototype: PCB for laboratory tests (**a**); and prepared for field tests with manually soldered NTC , normal mode helix antenna (NMHA) and battery (**b**); the RF performance is limited by the balun layout (a) and the proximity to the antenna of ferromagnetic crystal case and battery seal (b).

Early node prototypes were based on zero standby energy designs discussed in Section 2.2. Since their health status (QoS) cannot be observed, they were abandoned for a typical WSN node architecture: a microcontroller communicating with a radio device and powered by a primary cell, as shown in Figure 3.

The prototype was designed mainly to test the operation of the low-resource microcontroller and radio in a compact format, including the radio frequency (RF) performance, and to develop the node firmware, software and in-field communication protocol. Thus, the node printed circuit board (PCB)did not include the transducer, the power supply pads did not fit a standard battery size and it had just generic soldering pads for the antenna (all of these elements were still to be decided).

According to application specifications, the node prototype was primarily optimized for cost and size. In this regard, it is worth noting that it has significantly lower resources than a typical WSN node: the microcontroller is an ATtiny25 (2 KiB FLASH, 128 bytes SRAM, 128 bytes EEPROM, one analog to digital converter (ADC), one serial peripheral interface (SPI)); the radio for in-field communication is a TI CC1150 with a baseband modem (transmit-only for the 315-/433-/868-/915-MHz bands); and the external accurate resonator for the radio is a 27MHz quartz crystal. The microcontroller uses its internal oscillators (8MHz main clock and 128 kHz watchdog clock) to reduce the external components, the wake-up time and the energy consumption.

The average standby current of the sensor node is about 4.7 μA, largely due to the watchdog timer. It samples the temperature once per second with an NTC transducer (discussed in Section 5.1), keeping both the microcontroller and the transducer active for less than 0.05% of the time. The former consumes about 600 μA in active state and the latter a few tens of microamperes; thus, their contribution to node average energy consumption is negligible. The transmission of one packet consumes about 27 mA for less than 0.3 s. Thus, in normal operation (sending one heartbeat packet per hour, as discussed in Section 4.1), the average current consumption is less than 7 μA, and the node theoretical lifespan with a 1 Ah 1/2-AA size lithium battery exceeds 16 years.

### 4.3. Gateway Node Design

**Figure 4:**
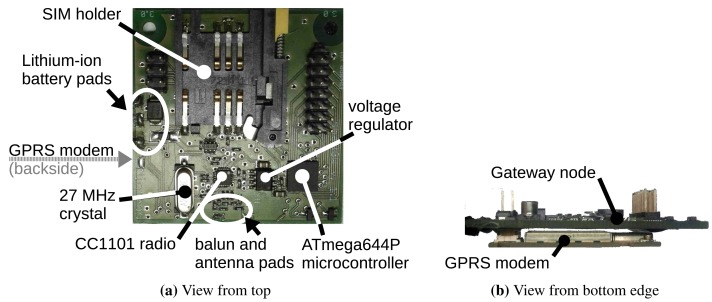
Top view (**a**) and bottom-edge view (**b**) of the gateway node prototype (50 × 50 mm). The layout of the balun (a) limits the RF performance and the battery charger of the modem is not efficient for energy harvesting.

The gateway prototype shown in Figure 4 is an evolution of an earlier prototype using a small ATmega324P microcontroller (32 KiB FLASH, 2 KiB SRAM, 1 KiB EEPROM) that was continuously relaying the data from the sensor nodes to the application server using the on-board general packet radio service (GPRS) modem, with almost no power management. It helped with testing the sensor node and server prototypes and with developing the modem driver and the recovery mechanisms from various GPRS connection errors.

The evolution shown in Figure 4 uses an ATmega644P microcontroller (64 KiB FLASH, 4 KiB SRAM, 2 KiB EEPROM), which has more SRAM and allows more extensive sensor node message aggregation. It uses a TI CC1101 transceiver for in-field communications, compatible with the sensor node transmitter. The GPRS modem (on the back side of the board; see Figure 4b) is a Siemens TC65i with an embedded TCP/IP stack and a charge regulator for lithium-ion batteries, which can be used for energy harvesting.

It is worth noting that the gateway node prototype is cost- and energy-effective, since it uses hardware resources that are typical for a low-end WSN sensor node (except for the GPRS modem).

## 5. Platform Preparation for the Field Test

While the platform prototype was still in the early stages of development, it became unexpectedly necessary to prepare all of its components (the sensor and gateway nodes and the server) for an extended two and a half month field test (end of June to early September). The test was set to start in less than two months using 50 sensor nodes and two gateways deployed in an olive tree field in the south-west of Greece. In the following, we will present how we selected, developed and assembled in this short time a platform prototype with the most important features to test in the field. We will also show how we used the results and experience from the test to refine and enhance platform functionality and reliability.

The time to field test was too short to design, produce and test new sensor nodes, and we decided to adapt the functionality and ruggedize the node prototypes designed for laboratory tests (described in Section 4). As expected, this adaptation has led to several issues during the test, but also fostered a thorough analysis of the platform operation before, during and after the test, ultimately helping us to significantly improve its reliability and cost, as we will show below.

Because the test field was located far away (nearly 2000 km), on-site maintenance would have been costly and time-consuming. On the other hand, since the components of the platform were not developed with the quality necessary to withstand an extended environmental exposure in field, we expected to encounter hardware issues during the test.

Thus, we made a development priority to add the necessary monitoring features to collect enough data about defects and anomalies that may manifest during the test, in order to understand their causes and effectively help with improving the future versions of the platform. Most of these features would be needed anyway, since QoS control is always important for long-term outdoor environmental monitoring platforms, especially when monitoring for potentially dangerous events, like wildfires, as was discussed in Sections 3.1.3 and 3.2.3.

Based on the analysis in Sections 3.1.2 and 4.1, we chose a star network topology and a very simple communication protocol, because they can considerably simplify the sensor nodes: reduce the requirements for hardware resources, the size and complexity of the communication protocol stack, the energy consumption and the cost. All of these are important specifications for pervasive monitoring platforms and, most importantly, they simplify network deployment to a level where it can be efficiently done by application domain experts or low-skill workers.

**Figure 5:**
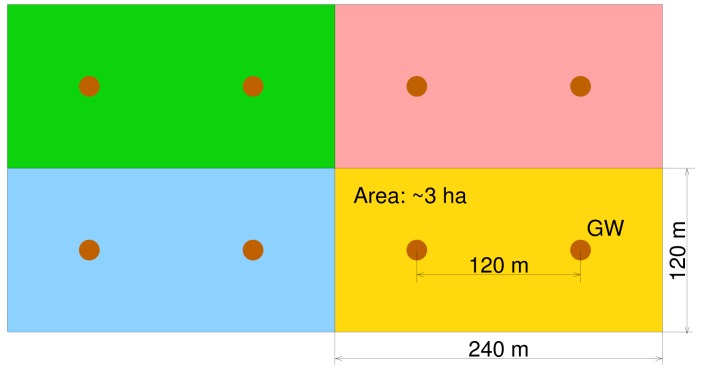
The network is made of independent cells with a star topology inside (the distances assume a 200m sensor communication range). The sensors in each cell are covered by two gateways, which can independently contact the server to ensure redundant field message aggregation and upload. Larger areas can be covered by joining elementary cells.

With the above considerations, we split the network into independent cells, as shown in Figure 5.

The sensors in each cell are distributed with the density defined by the domain experts based on terrain and vegetation characteristics (e.g., every 20 m) and are covered by two gateways. Each gateway can independently contact the server in order to ensure redundant field message aggregation and upload. Larger application areas can be covered by joining elementary network cells, as shown in Figure 5.

**Figure 6:**
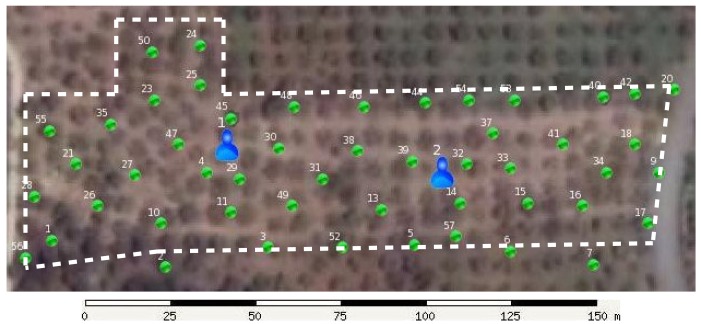
The test field is about is about 195 × 45m (main body). Trees appear as darker spots on the light-colored soil. There are two gateways and 50 sensor nodes (round dots; some are placed outside the field due to GPS positioning errors).

Although with a link budget of, e.g., 110 dBm, the communication range in the 433-MHz band can well exceed 10 km in optimal propagation conditions, the actual range in forest (or in the test field) conditions may be much shorter. Thus, the distances in Figure 5 assume a sensor communication range of 200 m, and the test field shown in Figure 6 of about 195 × 45m can be adequately covered by one cell.

This layout simplifies network deployment (presented in Section 6.1) and ensures full redundancy for field data collection, storage and communication to the server.

### 5.1. Preparation of the Sensor Node for the Field Test

**Figure 7:**
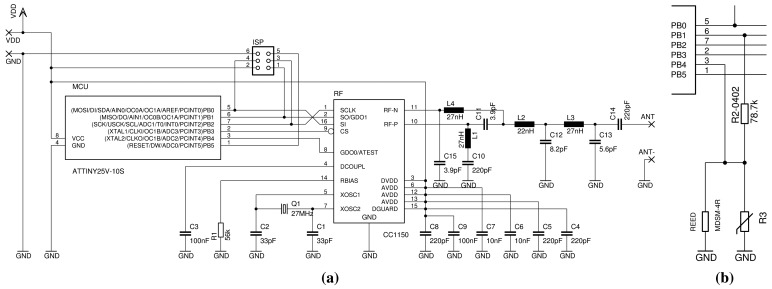
Schematic of the sensor node prototype for laboratory testing (**a**); issues fixed (**b**): (1) add a temperature transducer with an interface circuit and (2) add a means to switch the sensor to deployment mode.

The sensor node prototype described in Section 4.2 was designed for laboratory tests. It lacked several features for field tests, such as a temperature transducer, its interface circuit and a microcontroller input to read the transducer. Thus, the node schematic (shown in Figure 7a) was manually changed directly on the PCB, as shown in Figure 7b. R3 (an NTC) and R2 were connected as a voltage divider that was supplied only during the readings (to reduce node energy consumption) by reusing the SPI output pin PB1 as a software-controlled voltage source. At the same time, PB4 was reconfigured as an ADC input to read the transducer, cutting its connection to the radio module (which was needed only for tests).

These hardware and software changes allowed the sensor node to perform its main functions, monitoring the ambient temperature and notifying about abnormal high levels or too steep increases. The sensor nodes were programmed to measure the temperature once per second and to send specific alert messages when levels highly correlated with wildfires are detected.

The sensor node has to support also a fast and error-proof field deployment procedure, as discussed in Section 3.1.3. Even if the field test was using just a few dozen sensors, it was worth defining and testing from the beginning an effective field deployment procedure suitable for large applications, with hundreds or thousands of nodes that are deployed by personnel with no special WSN training.

These three items are especially important during the deployment of homogeneous sensors:

(1) the ID of the sensor node;
(2) the quality of the reception of the sensor node messages by the gateways;
(3) the field coordinates of the sensor node deployment spot.

The node deployment procedure should minimize human input, because the operators may only have limited training and time. Thus, the automation of the procedure can significantly reduce deployment errors and cost.

For an efficient field deployment, it is very important to be able to selectively address the node to be installed while other nodes are within comparable short distance (e.g., in the operator's backpack). Relying on the operator to input the node ID can be very time consuming and error prone, so an inexpensive (component cost and energy) near field communication with the node to install is necessary.

For this purpose, we added a reedswitch in parallel with the NTC (see Figure 7b), which can be activated using a magnet from a distance up to about 2 cm. When activated, the ADC will read 0V as the transducer output, which can be easily recognized, and temporarily switch the node to installation mode. In this mode, the node sends a few messages prompting the gateways in range to generate special acknowledgments that include the quality of the signal received from the node and the node ID. These data can be displayed on a handheld device to provide immediate feedback to the operator on the quality of the node installation position under evaluation. The handheld device can also determine the position of the node using an on-board GPS receiver and send all of the data to the server once validated by the operator.

This node installation flow requires minimum input from the operator, specifically:

trigger the deployment mode of the node by rubbing a magnet on its case. This is an intuitive and error-proof way to address the node under installation;
read on a handheld device the quality of the current deployment position;
tap a button on the handheld display to validate the deployment and send the data to the server.

Because of the short time available to prepare the field test, we implemented this flow only partially. We mounted the reed switches on the sensor nodes and changed the gateway protocol to provide the reception quality in the acknowledgments. The handheld device was implemented using a modified gateway that was displaying only the lowest reception level of the gateways in range on three LEDs.

During normal operation, the sensor node periodically tests the on-board temperature transducer given its importance for the detection of the fire. The node can detect short-circuits of the transducer (decoded as installation requests, as described above) and open-circuits. The node notifies to the gateways both faults in order to propagate them to the server which warns the operators about the fault.

Besides recognizing the extreme cases of short- and open-circuit, the selection of the temperature thresholds for alerts is very important. Low thresholds ensure timely alerts, but they also increase the sensor susceptibility to false alerts triggered by natural phenomena, such as sensor heating by direct exposure to sunlight. These considerations are especially important for fields with high solar exposure, as is the test field during summer time. After some testing, we decided that the fire alert threshold can be set to 60 °C and the threshold for warning of possible fire to a temperature increase rate of 1 °C/s.

A normal mode helix antenna (NMHA) was soldered to the antenna pad on the right of the PCB, as shown in Figure 3b. The NMHA is made of about 12 turns on an internal diameter of ϕ 10mm with a ϕ 1mm copper wire and has about a 21.5mm length. The number of turns and the length were experimentally tuned to maximize the RF radiation in deployment-like conditions.

**Figure 8:**
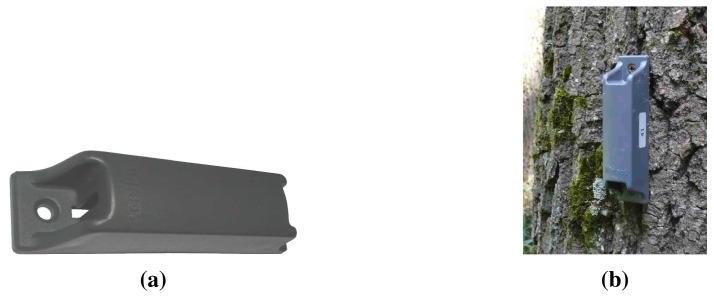
Sensor node package hosting the PCB, a half-AA battery and the normal mode helix antenna (NMHA) antenna. The aperture on the left improves the NTC thermal contact (**a**); fixed on trees with screws (**b**).

The sensor node package designed for the field test is shown in Figure 8. It holds the node PCB shown in Figure 8b with a half-AA battery soldered on top, an NTC transducer to the left and the NMHA antenna mounted axially to the right. The programming head on the left of the PCB was removed, and everything was sprayed with a protective resin against moisture and insects. The aperture on the left side of the package improves the thermal contact between the NTC and the ambient air. The node is mounted vertically on trees, at a height of 0.5 m, with the aperture pointing downwards for a good contact with the hot air rising from ground fires, which are notoriously difficult to detect using other techniques.

### 5.2. Preparation of the Gateway for the Field Test

To reduce the cost, the gateway node prototype for laboratory tests (described in Section 4.3) is implemented using resources that are typical for a WSN sensor node. The prototype can be used for the field test, although it is not optimized for low energy, as we will show below.

The resources of the microcontroller are sufficient for the gateway tasks. However, more SRAM would allow it to aggregate additional field messages, reducing the frequency of connections to the server and the energy consumption.

The transceiver for in-field communication is used to receive messages from sensor nodes and to communicate with peer gateways for health checks and to increase the availability of service, e.g.:

in case of failure of the GPRS link of a gateway:
  – it can send its field messages to peers, so that they forward them to the server;
  – send self-diagnostic data to the server through peers to provide the remote operators the necessary information for corrective actions;
failures of the field receiver would prevent the gateway from receiving hello messages from peers; thus, the gateway would eventually declare all peers “missing”; however, since the peers would regularly contact the server without reporting errors, this condition can be detected and appropriately flagged on the server;
failures of the field transmitter would prevent the peers from receiving the gateway hellos, which they will report to the server; the faulty gateway itself would not report any anomaly.

Gateways require much more energy than sensor nodes due to the continuous LPL of the sensor node channel (to support the random MAC) and energy consumption of the GPRS modem. Although the latter is turned off most of the time, the average energy consumption of the gateway is still too high to allow its long-term operation from a reasonably-sized primary cell. Thus, we installed solar panels on the gateway for energy harvesting, since the test field receives abundant sunlight.

**Figure 9:**
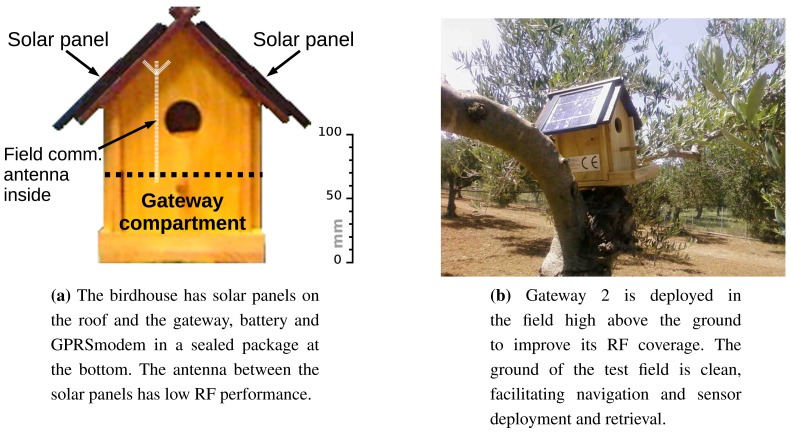
Gateway package elements (**a**) and field placement of Gateway 2 (**b**).

Considering the above, the gateway was embedded in a birdhouse, as shown in Figure 9a.

The solar panels for energy harvesting were placed on the roof, and the internal space of the birdhouse was divided horizontally into two compartments using a wooden separator. In the lower one were placed the gateway, the rechargeable battery and the GPRS modem in a sealed plastic package. The antenna of the GPRS modem was left inside the plastic package, because the test field had good cellular coverage. The antenna for in-field UHF communications, a λ/4 whip, was kept inside the birdhouse for aesthetic reasons. As expected, this position reduced the RF sensitivity, as we will discuss below.

### 5.3. Preparation of the Server for the Field Test

**Figure 10:**
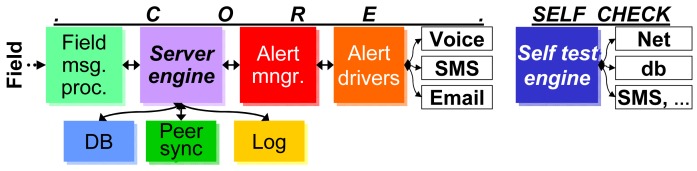
The application server structure is split into core services and a self-test engine.

The structure of the application server is shown in Figure 10 and has two major parts. One implements the core functions, such as listening on a socket for incoming connections from the gateways, decoding, saving and replicating field messages and alert management. The other implements the self- and external service checks, such as network connectivity, peer server, local database and SMS providers for alerts.

For the field test, the application server was updated to support the extensions of the gateway protocol, such as to send the receive acknowledgments to gateways to prevent field data loss, support for new message types for field device health semantics, session initiation and termination and time synchronization with the gateways. The user interface was extended to effectively display many sensor nodes with multiple health states, to improve responsiveness and usability and provide several operator authorization levels.

## 6. Field Test of the WSN Platform

Field deployment was scheduled on two days. On the first day, the field planning that was prepared in advance (remotely) was checked, followed by the actual field deployment. During the second day, the operation of the system was checked in the field for final tunings before commissioning to field test. Larger deployments may take more effort and time to complete, but they can follow the same sequence of operations.

### 6.1. Field Deployment of the WSN Nodes

Because the system was experimental and the field was rather small, we decided to monitor all communications between the nodes during field deployment from a fixed location near the field, using a modified gateway that was programmed to decode and display the messages as they were sent in the field. To detect abnormal behaviors during deployment and to improve the procedure, the meaning of these messages was manually compared with the state of the field displayed by the server, which was built using the field information received from gateways.

#### 6.1.1. Field Deployment of Gateway Nodes

The gateways were deployed in the locations shown in Figure 6. Gateways are always deployed before sensor nodes for star topologies to allow checking the connectivity of the nodes during installation.

While the sensor node deployment procedure described in Section 5.1 provides the means to check the quality of its connectivity within the network, no such provision was made for the gateways, because of the short time we had to prepare the field test. The assessment of the quality of a gateway deployment would include cellular coverage for the GPRS modem, light exposure for energy harvesting and the coverage of the sensor nodes in the field.

For this test deployment, we checked manually in the server log that the gateways were able to successfully connect. Furthermore, we monitored the field messages in real time for reports of success (or errors and retries) of the connection steps performed by the gateways: register with the cellular operator, access the GPRS service and open a socket with the server over the Internet.

The deployment places for the gateways were chosen so that one solar panel was roughly directed towards south or on the east-west axis, and the sunlight was reaching them without major obstructions. Figure 9b shows an overview of part of the test field and the deployment position of Gateway 2.

#### 6.1.2. Field Deployment of Sensor Nodes

Once the gateways were in place and operational, we started to deploy the 50 sensor nodes in the locations shown in Figure 6 with the procedure described in Section 5.1. Due to the limited time we had to prepare the field test, the deployment procedure was only partly automated. While the deployment mode of the sensor nodes can be quickly activated using a magnet, the operator would still need to manually record the node ID and position coordinates read from a handheld GPS. These operations proved to be time-consuming and error-prone and required us to make several passes through the field to recheck node IDs and positions. Moreover, as can be seen in Figure 6, some positions were still slightly out of the field (e.g., Sensor Nodes 2, 6, 7) due to the limited accuracy of the GPS receiver.

Using the node deployment procedure presented in Section 5.1 (only partly automated, as we have mentioned), we needed about 4–5 min to install one sensor node in a relatively simple field (see Figures 6 and 9b): the trees were well spaced and organized in rows; most ground vegetation and obstacles were cleared; and the GPS signal was received without the typical attenuation from dense tree crowns.

Nevertheless, it took us about four hours to deploy all 50 sensor nodes. This means that node deployment is generally effort intensive and can significantly increase the cost of larger applications.

### 6.2. Issues during Field Deployment

#### 6.2.1. RF Range Issues

Although the size of the test field was relatively small compared to the communication range in ideal conditions of about 17 km for the 433-MHz band with a 110 dBm link budget, we noticed during deployment that some nodes at more than 50–70m from gateways were received little or not at all.

Several design decisions reduced the communication range. First, the placement of the gateway antenna for field communications between the solar panels of the birdhouse (see Figure 9a) reduced its efficiency. We expected this, but aesthetic considerations prevailed, since we had not the time to measure the actual loss before the test. After the test, we measured about a 24–26 dBm loss, a penalty that led us to install external sockets for all aerials in the new birdhouse project.

After the test, we discovered two additional RF attenuation causes on the PCB of the sensor node. As shown in Figure 3b, the antenna is just a few millimeters away from two large ferromagnetic items: the battery side seal and the case of the quartz crystal resonator. The typical loss of the NMHA is about 6–8 dBm, but these two elements combined were adding about a 10 dBm loss. Thus, in the next version of the sensor node PCB, we will use a different case for the crystal resonator and move the battery away from the antenna to reduce its RF attenuation.

The layout of the baluns of both the sensor node and the gateway (shown in Figures 3a and 4a) was compacted to reduce the size of the nodes, size being especially important for the sensor node. One of the purposes of the node prototype was to determine the loss of the compact balun layout, which we determined after the test to be around 3 dBm. Thus, we accepted the larger size of a straight balun layout.

The package of the sensor node (shown in Figure 8) was introducing an attenuation of about 2 dBm, but this cannot be easily avoided.

Another attenuation cause was due to terrain geometry. The test field shown in Figure 6 had a very gently slope at the bottom-left corner, almost imperceptible to the eye. To increase the sensitivity to heat released by ground fire, we installed the sensor nodes low on the tree trunks, about 0.5m above the ground. The gateways, on the other hand, were placed higher in the trees to improve both energy harvesting from sunlight and RF reception from low-placed sensor nodes. However, Node 1 and, even more, Node 56 were attenuated too much by this small terrain undulation, which was easily discovered by the checks performed during deployment. Thus, we decided to install the affected nodes higher, at 1m from the ground. Nevertheless, this incident shows that even small terrain undulations may restrict the coverage of the gateways, especially for distant nodes, and this should be taken into account when choosing the positions of the gateways in the field. We also decided to develop a repeater node that can be deployed to improve the gateway coverage of the field.

In general, the deployment positions of the gateways on hilly terrain should be carefully analyzed during planning. Nevertheless, there can always be propagation issues that are discovered only during deployment (e.g., due to fading from multipath propagation or obstacles). Some of these issues can be fixed by changing the position of the sensors (a different position on the tree trunk, higher from the ground or on a nearby tree). In other cases, we can always add a gateway to cover these areas or, even better cost-wise, a repeater device.

#### 6.2.2. Temperature Issues for Sensor Nodes

As the deployment of the sensor nodes progressed and the Sun approached the zenith, some of the sensor nodes waiting to be deployed started to send fire alerts. Apparently, this was because the nodes were transported in a dark color backpack which excessively heated them while lying idle in direct sunlight (the test field had only a few patches of shadow, as can be seen in Figure 9b).

Rising the alert threshold of the sensor nodes would avoid false alerts, but it would also reduce the detection sensitivity of fires, delaying the alerts. Since timely alerting is an important feature of the system, we chose to revise the transport and deployment procedure to prevent sensor node overheating (e.g., by using lighter colors for packs during transport and avoiding extended exposure to sun heat).

### 6.3. Long-Run Test of the WSN Platform

After all nodes were deployed and the operation of all platform components was checked, the field test started for two and a half months.

#### 6.3.1. Gateway Issues during the Long-Run Test

**Figure 11:**
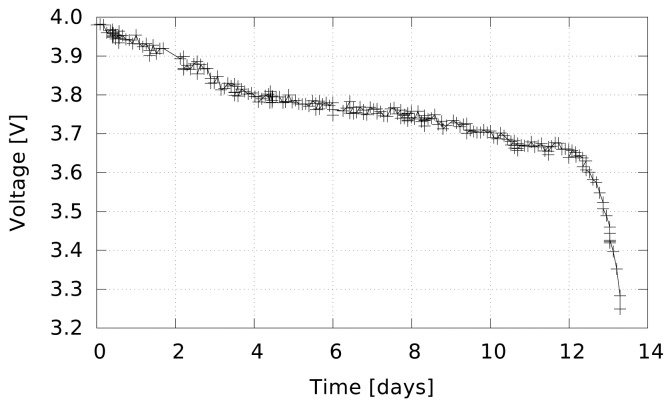
Lithium-ion battery voltage discharged by a gateway that contacts the server every 15 min to transfer 50 messages. It has an almost flat plateau, just below 3.8V.

Post-deployment checks missed a very important fault. The battery charging circuit of the GPRS modem of Gateway 2 failed during deployment and was not charging the battery. We discovered this too late, after we left the field, because we were monitoring the battery voltage (measured by the GPRS modem), and the potential *vs.* charge relationship, shown in Figure 11, has an extended, almost flat plateau that makes it difficult to determine changes in battery charge from voltage readings.

The battery charger of the modem of Gateway 2 may have failed because of overheating during deployment due to a combination of the following three factors. First, Gateway 2 was the first deployed, thus it received more sun heat, which increased its internal temperature. Second, it received and forwarded to the server most of the false fire alerts generated by the sensor nodes, which additionally increased the temperature of the modem. Third, since the solar panels were well illuminated, they were charging the battery, thus increasing the temperature of the charging circuit even more.

As Gateway 2's battery progressively discharged, we monitored the effectiveness of the built-in in-field redundancy. The GPRS modem does not work below 3.2V, but the rest of the gateway (microcontroller and field radio) operates until the battery is completely discharged (lithium-ion batteries stop working around 2.7 V). When battery voltage dropped below the low threshold of the GPRS modem and it ceased to work, Gateway 2 continued to operate as a repeater, forwarding to its peer the messages received from the sensor nodes in its range. Gateway 1 aggregated them with its own messages and sent them all to the server, maintaining the redundant coverage of field nodes, as expected. When Gateway 2 eventually stopped working completely, Gateway 1 detected the new fault condition and notified the operators through an error message sent to the server.

Another temperature-related issue was due to the limited range allowed for charging the lithium-ion batteries, 0–45 °C. Open nature temperatures can exceed this range, thus blocking battery recharging and, eventually, reducing its lifetime. These limits make lithium-ion rechargeable batteries less suited for open space environmental monitoring.

**Figure 12:**
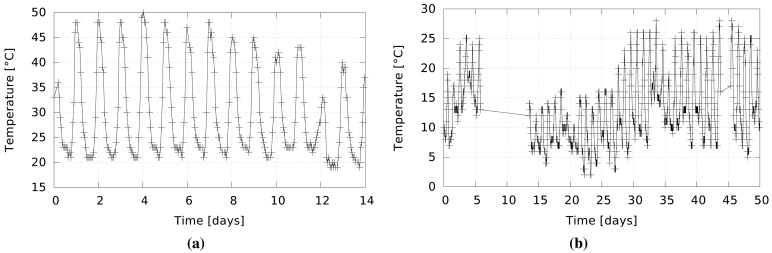
Gateway temperature can exceed 45 °C, the maximum allowed for lithium-ion battery charging (**a**), while the internaloscillator drift can break the synchronous serial communication with the GPRS modem at low temperatures (Days 6–13 and 43–45) (**b**).

During our field test, high daytime ambient temperature combined with heat from gateway direct sun exposure and heat released during GPRS communication and battery charging were often blocking battery charge during the central hours of hot days. Figure 12a shows how the internal gateway temperature met or exceeded 45 °C on most days over a two-week period. However, this did not significantly affect the operation of the gateway, since hot days are typically also very sunny, thus providing sufficient energy for recharging. Nevertheless, heat dissipation was not adequate for this gateway prototype. The gateway package should be adapted to the climate of the monitored field.

Figure 12b shows how low temperature may break the synchronous serial communication between the microcontroller and the GPRS modem (approximately between Days 6–13 and 43–45 in the figure). However, since these failures correlate loosely with temperature, we could not rule out other causes, such as condensing humidity inside the gateway or inside the GPRS modem, until we reproduced the ambient conditions using a climatic chamber after the field test. The log collected locally on the affected microcontrollers indicated serial communication errors that were resolved by recalibrating the internal oscillator of the microcontroller from an accurate reference (e.g., a scaled-down frequency of the field radio crystal resonator).

#### 6.3.2. Sensor Node Issues during the Long-Run Test

Thermal expansion mismatch between the PCB and the NTC leads generated repeated mechanical strains due to cyclic temperature variations of 15–20 °C between day and night. These eventually led to fatigue cracking of NTC solder joints on some sensor nodes, which, in turn, led to intermittent contacts, especially during morning and evening hours, when the temperature changed most rapidly. The sensor node decoded the imperfect contacts as fast ambient temperature changes, and notified these right away to the gateways as wildfire warnings (see Section 5.1). The gateways handled these warnings the same way as fire alerts, forwarding them immediately to the server to promptly notify the operators.

Since the GPRS modem is one of the highest energy drains of the gateway, keeping it powered up for a few hours in the morning and evening every day to forward these warnings eventually consumed more energy than the harvesting capacity of the solar panels and discharged the battery of the only gateway left. Its failure required a maintenance operation to restore both gateways to full functional state and to replace the faulty sensor nodes.

Such sensor node faults can happen also in large deployments that should run unattended for many years. Thus, the system should be able to exclude the network effects of faulty nodes before they can create larger problems that may lead to (partial) loss of service. For this purpose, we decided to provide the gateways with a list of sensor node IDs that should be ignored and which can be set remotely by the operators when needed. Such a list would have helped us avoid a costly field trip for maintenance during the test.

Last, but not least, all systems powered by harvested energy (which may fluctuate widely reaching very low levels) should have brown-out detection (BOD) enabled to avoid entering non-functional states when the battery voltage is too low. Because the BOD was not enabled on the gateway prototypes, Gateway 1 failed several times to return to normal operation after periods of insufficient energy.

## 7. Conclusions and Future Work

*In situ* pervasive environmental monitoring for wildfires has important advantages, but the relatively high area cost may limit its use. We have shown in this paper several domain-specific platform optimizations that reduce the cost and increase its economic viability.

Sensor node hardware and a communication protocol optimized for application requirements can exceed 10 years of life time using a small, low-cost primary cell. Long node life significantly reduces the cost of the scheduled maintenance of the network, which can be important for large networks. The nodes and the communication protocol also speed up field deployment. The deployment procedure prevents most human errors, significantly reducing the cost, especially for networks with a large number of nodes.

Field experiment preparation and unfolding highlighted several aspects that can improve the cost and reliability of the network components, their deployment, operation and maintenance.

Long-term direct node exposure to climatic conditions increases the failure probability. Additionally, large networks are more likely to experience node failures. As we have seen during the field experiment, failed nodes may adversely affect network operation or strain the resources of the gateways in range, increasing maintenance cost. Thus, it is important to increase node and network robustness during design, production testing, functional and self-test specifications and run-time monitoring.

As we have shown, node hardware design and production testing were not adequate for the field experiment and led to several node failures. Accumulation of mechanical stress should be reduced by design and during production, since it compounds, e.g., with the stress from daily and seasonal temperature variations or unforeseen mechanical interaction with wild animals. Manual soldering should be avoided, since it can inadvertently create strains in solder joints in the interaction with component leads.

Faulty nodes should be detected as soon as possible to reduce production costs, e.g., by running specific self-test programs at the end of the production line.

Lithium primary batteries have shelf lives in excess of 10 years. Compared to ambient energy harvesting techniques suitable for open nature, primary cells can reduce node cost, increase reliability and reduce node deployment complexity. These cells can supply very low power nodes cost effectively and maintenance free for more than 10 years, a life time suitable for many WSN applications.

Brown-out detectors may consume too much energy and should not be needed for long lifetime nodes with soldered batteries. However, it is important to reset the nodes right after the battery is soldered to avoid leaving them in abnormal states that may excessively drain the battery.

Sensor node functions and a communication protocol carefully tailored to the application requirements can bring important cost reductions. For instance, transmit-only nodes in star topologies can reduce the protocol stack and relax the accuracy requirements for on-node timing. In turn, these reduce microcontroller resources and energy consumption, significantly lowering the cost of components, battery and node maintenance. However, without a receiver, the nodes cannot create mesh topologies to improve network robustness and to reduce transmit power. Thus, the cost and benefits of such optimizations should be evaluated in the context of the specific application or application class.

Periodic node self-testing and automatic error recovery increase the reliability and the QoS of the monitoring platform, reducing maintenance requirements and cost. In case of critical failures (e.g., failing contact with the NTC), the nodes may disable themselves after reporting the fault to avoid reporting false events or impairing the operation of the network or the gateway nodes, as we have witnessed during the field experiment.

Small sensor node physical dimensions and protection from environmental conditions may reduce the RF efficiency in several ways. For instance, the antenna may end up too close to large ferromagnetic bodies, like the battery side seal or the case of the quartz crystal resonator in our experiment. While we could use a different case for the crystal resonator to avoid changing the node geometry, we had to increase node length to leave enough distance between the antenna and the battery seal. Furthermore, protecting the antenna inside the node package reduced its efficiency by 2 dB, but we found no cost-effective way to avoid it. However, component cost is less important for gateways, and we plan to install their antennas clear of the package or solar panel shielding, which have markedly reduced their performance during the field test.

Changes in gateway behavior in the case of failures (e.g., GPRS link or field radio receiver failures) need to be detected at the gateway or network level and reported to network operators to schedule corrective actions. Remote configuration or reprogramming should be available, at least for the gateways, in order to be able to remotely add functions, correct errors or protect the network against occasional faulty node behavior. As we have seen during the field experiment, this feature would have spared us an unscheduled maintenance, whose cost can be important for remote fields or large networks.

Nodes that store field messages before forwarding (e.g., gateway nodes) should implement suitable in-field redundancy to avoid loss in case of self-reset or failure. Additionally, we plan to implement a save-restore function on gateways to preserve node data across watchdog-triggered resets.

Gateway nodes handle higher energies, which can generate significant heat, both during harvesting and consumption peaks (e.g., during GPRS data connections with the server). Thus, as we have seen during the field experiment, an adequate thermal dimensioning considering the climate and the internal and external heat sources is important. For some applications, we may consider other energy accumulation techniques instead of the widely-used lithium-ion batteries, which have limited charge-discharge cycles and temperature restrictions for charging and operation.

We also consider redesigning the power supply of the gateways to automatically switch to a primary cell whenever the harvested energy reserves are too low, to increase the node QoS. Unlike the sensor nodes, which are reliably supplied by the battery soldered on-board, the energy reserves of the gateways can fluctuate due to unpredictable harvesting and consumption patterns. Moreover, the rechargeable batteries can be changed when they loose storage capacity. Hence, the use of the embedded brown-out detector can considerably improve gateway stability.

The field experiment showed us that the deployment of the network can be very time-consuming, even for networks with just a few dozens of nodes. Thus, the procedure should be simple and automated as much as possible to be accessible to domain operators, to reduce human errors and cost. Nevertheless, it should allow one to thoroughly check the quality of node installations, since RF propagation in open nature can be unpredictable, not the least due to small and difficult to notice terrain features. We plan to improve the user interface of the handheld installation device, automate position collection and data reporting and extend it to the installation of the gateways. For the latter, it should assess cellular coverage for the GPRS modem, light exposure for energy harvesting and the coverage of the sensor nodes in the field. We also decided to develop a repeater node that can improve gateway coverage of the field in difficult propagation conditions.

WSN platform design, development and maintenance has a high cost, which can be recovered faster if the platform can be reused for several related application domains. While the sensor nodes do not have sufficient resources to support an IP-based protocol, the gateways are IP-enabled, albeit they are sporadically connected. The server, however, is always accessible over the Internet, and in future versions, it can implement suitable interfaces (e.g., based on OGC^®^ Sensor Web Enablement [91–94]) to facilitate the discovery and access to sensor and field data by authorized IoT applications.
